# Role of the Microbiota and Antibiotics in Primary Sclerosing Cholangitis

**DOI:** 10.1155/2013/389537

**Published:** 2013-10-22

**Authors:** James H. Tabibian, Jayant A. Talwalkar, Keith D. Lindor

**Affiliations:** ^1^Division of Gastroenterology and Hepatology, Mayo Clinic, 200 First Sreet SW, Rochester, MN 55905, USA; ^2^Executive Vice Provost & Dean, College of Health Solutions, Arizona State University, 550 North 3rd Street, Phoenix, AZ 85004, USA

## Abstract

Primary sclerosing cholangitis (PSC) is an idiopathic, progressive, cholestatic liver disease with considerable morbidity and mortality and no established pharmacotherapy. In addition to the long-recognized association between PSC and inflammatory bowel disease, several lines of preclinical and clinical evidence implicate the microbiota in the etiopathogenesis of PSC. Here we provide a concise review of these data which, taken together, support further investigation of the role of the microbiota and antibiotics in PSC as potential avenues toward elucidating safe and effective pharmacotherapy for patients afflicted by this illness.

## 1. Introduction

 Primary sclerosing cholangitis (PSC) is a chronic, fibro-inflammatory, cholestatic liver disease of unknown etiopathogenesis that affects children and adults worldwide [1–3]. PSC leads to end-stage cirrhosis, represents a major risk factor for cholangiocarcinoma (CCA), and carries a median liver transplant- (LT-) free survival of approximately 12 years [4–6]. Despite clinical trials of over fourteen different pharmacologic agents, including but not limited to various immunosuppressants and antifibrotics, medical therapy for PSC has yet to be established [2]. Unlike in other cholestatic liver diseases (e.g., primary biliary cirrhosis), treatment with ursodeoxycholic acid (UDCA) has not been clearly shown to have a beneficial effect in PSC [7]. In addition, high doses of UDCA may be associated with significantly greater incidence of adverse outcomes [8]. Due to its progressive, idiopathic nature and lack of medical therapy, PSC remains a leading indication for liver transplantation in northern Europe and the United States despite its relatively low prevalence (0.5–1.5 per 10,000) [9, 10]. Although operative treatment with liver transplantation (LT) is effective for PSC, it is only performed in specialty centers and in select patients; furthermore, even in suitable LT candidates, PSC and CCA can recur post-LT [11–13]. Therefore, given the morbidity and mortality of PSC and the challenges associated with operative treatment, safe and effective pharmacotherapies are critically needed. 

 PSC is now generally accepted as being a heterogeneous, pathogenically complex disease, with genetic, immunologic, environmental, and other potential factors being involved [2, 14]. There are now several lines of evidence suggesting that one such important and ostensibly modifiable etiopathogenic factor is the microbiota, particularly enteric bacteria. Here we provide a concise review on the molecular/cellular, translational, and clinical data regarding the possible role of the microbiota in the pathogenesis of PSC and as a target of pharmacologic therapy for patients afflicted with this disease. 

## 2. Primer on the Relationship between PSC and the Gut

**Figure 1:**
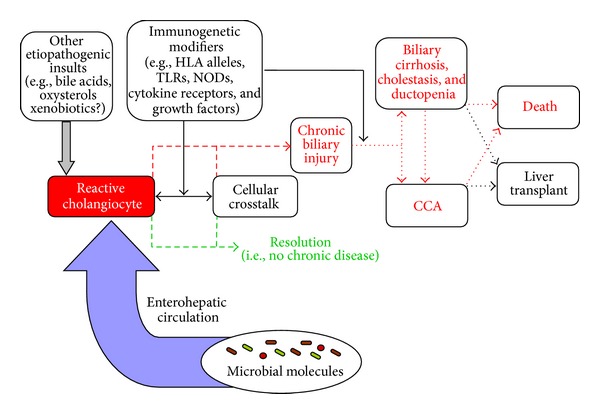
Proposed conceptual model of primary sclerosing cholangitis (PSC) etiopathogenesis. Cholangiocytes exist in a microenvironment abundant in potential etiologic mediators of cellular insult and activation, including microbial as well as nonmicrobial molecules. Whether PSC etiopathogenesis is related to increased exposure to constitutive molecular mediators of injury (e.g., through the enterohepatic circulation), alterations in the types of these mediators (e.g., due to enteric microbial dysbiosis), and/or an aberrant resident (e.g., cholangiocyte, hepatocyte) or recruited (e.g., lymphocyte) hepatic cell response remains uncertain. Immunogenetic factors can modify these variables and thus play a role in modulating initiation and progression of biliary injury in PSC. CCA: cholangiocarcinoma; HLA: human leukocyte antigen; NOD: nucleotide-binding oligomerization domain receptor; TLR: toll-like receptor.

 The association between PSC and the gut, most notably inflammatory bowel disease (IBD), was first reported over four decades ago [15, 16]. The geoepidemiology of IBD among patients with PSC is heterogeneous; for example, an estimated 75% of Western patients with PSC are (or become) codiagnosed IBD (the majority of whom have ulcerative colitis, UC) [1, 17] as compared to only 23% in some cohorts of Japanese patients [18]. The PSC-IBD association is thought to be related at least in part to the increased intestinal permeability observed in some patients with IBD [19–21] as well as the direct anatomic link between the gut and the liver, that is, the enterohepatic circulation [14, 22]. Although the putative gut-derived trigger(s) of hepatobiliary pathobiology in PSC has not been determined, microbial metabolites or products (i.e., pathogen-associated molecular patterns, PAMPs) such as lipopolysaccharide (i.e., endotoxin, LPS) and peptidoglycan (i.e., a bacterial cell wall polymer, PG) have been proposed as likely candidates [23, 24]. This forms the basis of what has been referred to as the “leaky gut” or “PSC microbiota” hypotheses (the latter not emphasizing a need for increased intestinal permeability given some patients with PSC who have no detectable bowel disease and normal intestinal permeability) [23, 25, 26]. Indeed, portal bacteremia and bacteriobilia have both been described in patients with PSC-IBD [27, 28]. However, the extent to which the association between PSC and IBD is due to (i) increased enterohepatic circulation of PAMPs, (ii) abnormal PAMPs (e.g., as a result of enteric microbial dysbiosis, as seen in IBD) [29–31], or (iii) an aberrant or hyperreactive cholangiocyte and/or innate immune response (i.e., as a result of “immunogenetic susceptibility” [24, 32, 33]) to essentially normal levels and repertoires of enterohepatically circulated PAMPs (or other potential gut-derived molecules) remains a key unknown; given the heterogeneity of PSC, it is possible that any of these three possibilities may be operative in a given patient (Figure 1) [2, 24].

## 3. Findings from Animal Models Supporting the Gut-Hepatobiliary Disease Relationship

 Several lines of experimental evidence from animal models demonstrate that enteric dysbiosis and/or administration of bacterial antigens can lead to hepatobiliary inflammation with various features of PSC. Lichtman et al. were the first to conduct a number of elegant studies in the early 1990s demonstrating this relationship. For example, rats with small bowel bacterial overgrowth as a result of surgically created self-filling jejunal blind loops were found to have abnormalities of the extrahepatic and intrahepatic bile ducts that resembled PSC both histologically and cholangiographically, similar to the abnormalities found in the multidrug resistant 3 (mdr2) knockout mouse [44] (currently among the most widely-utilized murine models of PSC) [45]. Interestingly, treatment of these rats with mutanolysin, an enzyme which breaks the *β*1-4 linkage of N-acetylmuramyl acid and N-acetylglucosamine inherent to PG, but not with UDCA, prednisone, methotrexate, or cyclosporin A, resulted in a significant decrease in plasma aspartate aminotransferase and liver histology scores [46]. Extending these findings, a subsequent study showed that intraperitoneal injection of rats with PG resulted in (micro)cholangiographic irregularities of smaller intrahepatic bile ducts and larger ductules, focal areas of narrowing and fusiform sacculations, and histologic evidence of bile duct destruction [47]. Another group found that rectal administration of N-formyl L-methionine L-leucine L-tyrosine, a chemotactic peptide produced by *E. coli* which, after entering the enterohepatic circulation, is secreted into bile by hepatocytes, resulted in a mixed inflammatory hepatobiliary infiltrate with predominantly small duct cholangitis [48].

 In a more recent study, it was demonstrated that repeated inoculation of Balb/c mice with *S. intermedius* resulted in nonsuppurative cholangitis and production of antibiliary epithelial cell and antinuclear antibodies, as seen in a considerable proportion of patients with PSC [49, 50]. In addition to the animal models described above, several other infectious models, including but not limited to *C. parvum*-inoculated mice, demonstrate at least some biochemical, histologic, and/or cholangiographic features of PSC, as reviewed in more detail elsewhere [51].

 Although these models (and for that matter, no toxin-induced, knockout, or other model) do not specifically recapitulate the entirety of findings characteristic of PSC, they do provide a premise which supports the notion that hepatobiliary disease in PSC may be modified, if not caused, by aberrant microbial molecule-host interactions. This premise, together with clinical or human cell line observations, as will be reviewed below, has provided impetus for a growing number of clinical studies of antibiotics in patients with PSC. 

## 4. Human Tissue-Based Translational Studies Supporting the PSC Microbiota Hypothesis

 Given that PAMPs such as LPS, PG, and other microbially derived molecules (e.g., lipoteichoic acid) have been proposed as possible contributors or drivers of hepatobiliary pathobiology in PSC, it is noteworthy to highlight the body of evidence that PAMPs can be directly detected by and induce signal transduction in cholangiocytes [24, 52–56]. Indeed, cholangiocytes are immunologically active cells that express pathogen recognition receptors, including nucleotide-binding oligomerization domain proteins (NODs) and all known toll-like receptors (TLRs) [54, 55]. Binding of microbially-derived and other immunoactive ligands (e.g., oxysterols) to these receptors results in signalling through a variety of adapter proteins and pathways that can culminate in the activation of profibroinflammatory transcription factors and ultimately hepatobiliary fibrosis and inflammation [24, 56]. As mentioned before, however, the key PAMPs involved in activating such pathways in PSC have yet to be delineated, and it remains unknown whether they elicit disease due to increased abundance, abnormal moieties/epitopes (e.g., of noncommensal, pathogenic bacteria), exaggerated host responses, or a combination.

 Several intriguing observations have been recently made in this regard. The first is that cultured cholangiocytes isolated from PSC liver (as well as peripheral blood mononuclear cells [57]) have been shown to exhibit persistent hypersensitivity (i.e., impaired immune tolerance) to pathogen recognition receptor agonists (e.g., LPS) [58]. In addition, increased TLR and NOD protein expression as well as activation of the MyD88/IRAK adapter protein signalling complex in PSC cholangiocytes have been described [58]. Of note, circulating antibiliary epithelial cell antibodies, an area of research that has been perhaps incompletely explored in the pathogenesis of PSC, may account for the induction of increased TLR expression in PSC cholangiocytes [50]. The second intriguing observation is that not only do patients with PSC exhibit bacterobilia [28] and detectable levels of 16s ribosomal ribonucleic acid (rRNA) in bile [59, 60], but also cholangiocytes in PSC liver sections (but not normal livers) have been shown to accumulate LPS [61], which is secreted into bile in a bioactive form [62, 63]. It should be noted, though, that it remains unknown to what extent bacterobilia or biliary 16s rRNA is the result of previous biliary interventions (e.g., endoscopic retrograde cholangiography) that occurred after PSC had already developed. The third observation is that genome wide association studies have found new PSC risk loci (aside from the human leukocyte antigen complex) related to immunoregulation and immune-mediated disease [64, 65]. One such example is fucosyltransferase-2, which has been shown to influence the microbiota, affect susceptibility to microbial infection, and be associated with IBD (specifically Crohn's disease) [65–67]. More translational studies are needed to determine the effects of both PAMPs and immunogenetic susceptibility on cholangiocyte activation, signaling, and their bearing on PSC pathogenesis.

## 5. Clinical Experience with Oral Antibacterial Agents in PSC

Since the publication of the initial case series of antibiotics in PSC in 1959 (at that time referred to as “chronic pericholangitis”) [36], a number of studies of antibiotics in PSC have been published, the majority being within the past 10 years [2, 68]. Three of these were prospective clinical trials (Table 1(a)): in the first of these three, Färkkilä et al. [34] recruited 80 patients and randomized them to 36 months of treatment with UDCA (15 mg/kg/day) plus metronidazole (*n* = 39) or UDCA alone (*n* = 41) in a double blind manner; the authors found a significant improvement in serum alkaline phosphatase (ALK), Mayo PSC risk score, and histologic stage and grade as well as a trend toward less cholangiographic progression in the UDCA plus metronidazole group after 36 months of treatment [34]. In the second clinical trial, Silveira et al. [35] conducted an open label pilot study wherein 16 patients with PSC were treated with minocycline for one year; although a quarter of patients withdrew from the study (majority due to adverse effects), those who continued minocycline treatment experienced a significant reduction in serum ALK and a trend toward a significant reduction in aspartate aminotransferase and Mayo PSC risk score. In the third and most recent clinical trial, Tabibian et al. [25] conducted a phase II, double blind, randomized pilot study of vancomycin and metronidazole. Thirty-five patients with PSC were randomized into 4 groups: low-dose vancomycin, high-dose vancomycin, low-dose metronidazole, or high-dose metronidazole. Although individual responses were variable, a significant decrease in ALK at 12 weeks (the primary endpoint) was evident in the low- and high-dose vancomycin groups; furthermore, 2 patients in the low-dose vancomycin group experienced normalization of ALK. Although patients in all 4 groups experienced significant improvements in at least one of the secondary endpoints (serum total bilirubin, C-reactive protein, Mayo PSC risk score, and pruritus), adverse effects were more frequent in the metronidazole groups. Thus, the authors recommended (low-dose) vancomycin for further investigation, ideally in a larger, longer-term, placebo-controlled trial [25]. 

 Aside from these three clinical trials, there have also been several case series and reports of oral antibacterial therapy in PSC (Table 1(b)) [27, 36–43]. Among the most notable of these is a case series by K. L. Cox and K. M. Cox of three pediatric patients with PSC and IBD who experienced normalization of liver tests and resolution of symptoms with oral vancomycin treatment [39]. In a more recent prospective series from the same group, 14 pediatric patients with PSC and IBD were treated with oral vancomycin for 54 ± 43 months [43]; the authors noted normalization or significant improvement in serum liver tests, erythrocyte sedimentation rate, and clinical symptoms in nearly all patients. In addition, when vancomycin treatment was discontinued, there was recurrence of clinical symptoms and an increase in liver enzymes in several patients, and retreatment again resulted in normalization of liver enzymes [43]. Of note, there have also been a case report and a small randomized trial of probiotics in PSC which yielded conflicting results [69, 70]. More recently, there has also been a case report of post-LT PSC successfully treated with oral vancomycin [71].

 Although antibiotic trials thus far have been generally small and few in number, taken collectively, their results are favorable and encourage further studies of antibiotics as a potentially safe, effective, and convenient therapy in PSC.

## 6. Conclusions and Future Directions

 The body of basic, translational, and clinical data supporting the PSC microbiota hypothesis continues to grow, and with the ever-evolving developments in molecular, cellular, and bioinformatic techniques, it is likely that more insights will be gained. Until then, and before oral antibiotics can become an established therapy for patients with PSC, the following questions and considerations remain.

1. What is the relative contribution of abnormalities in (i) the number or type of microbially-derived molecules as compared to (ii) the host response (i.e., immunogenetic susceptibility)?
2. What is the mechanism of potential therapeutic action of antibacterial agents on PSC? Is it, for example, related to (i) a direct effect on bacterial (and/or other microbial) load or diversity, (ii) secondary changes in the type or amount of bacterial components or metabolites (e.g., LPS and secondary bile acids, resp.) in the gut and/or in the enterohepatic circulation, or (iii) nonantimicrobial effects, for example, anti-inflammatory or immunoregulatory effects, such as those ascribed to minocycline and vancomycin, respectively [35, 72]?
3. Which PSC patients benefit most from antibiotic therapy, and what are the relevant modifiers (e.g., IBD status, age, and stage)? [2]
4. What antibiotic and dosing regimen (e.g., daily versus interrupted/alternating) will theoretically provide the most therapeutic effects while minimizing adverse effects such as antibiotic resistance?
5. Is it time for, and who will design, conduct, and fund, a larger, longer, randomized (i.e., phase II/III) clinical trial of antibacterial therapy in PSC?

## Figures and Tables

**Table 1a:** (a) Clinical trials of antibacterial treatment in primary sclerosing cholangitis

Drug (reference)	Year	*n*	Antibiotic dose	Months of therapy	Change after therapy		
					ALK	AST	ALT
Metronidazole (+UDCA) [34]	2004	39	600–800 mg/day	36	−52.4%	−41.0%	−67.9%
Minocycline [35]	2009	16	200 mg/day	12	−19.7%	−2.8%	NA
Vancomycin or metronidazole [25]	2013	18	Vancomycin 125 or 250 mg qid	3	−42%	−22%	NA
		17	Metronidazole 250 or 500 mg tid	3	−10%	−9%	NA

**Table 1b:** (b) Case series and reports of antibacterial treatment in primary sclerosing cholangitis

Drug (reference)	Year	*n*	Antibiotic dose	Months of therapy	Change after therapy		
					ALK	AST	ALT
Tetracycline [36]*	1959	5	500 mg/day	1–10	−45%	−60%	−45%
Tetracycline [27]^†^	1965	5	500 mg/day	48 (mean)	−21%	NA	NA
Metronidazole [37]	1983	1	800 mg/day	0.25	NA^‡‡^	NA^‡‡^	NA^‡‡^
Sulfasalazine (+UDCA) [38]^††^	1998	2^‡^	—	30 and 45	−79% −35%	−38% −87%	−70% −95%
Vancomycin [39]	1998	3^‡^	375–1000 mg/day	9 (mean)	NA	NA	−89%
Sulfasalazine (+UDCA) [40]	2002	1	50 mg/kg/day	37	NA	NA	−92%
Sulfasalazine [41]	2006	1	2–4.5 g/day	24	−74%	NA	−84%
Azithromycin (+UDCA) [42]	2007	1	500 mg/day, 3 days/week	5	−72%	−31%	−33%
Vancomycin [43]	2008	14^‡^	50 mg/kg/day	54 ± 43	NA	NA	−78%

Key: ALK: alkaline phosphatase, AST: aspartate aminotransferase; ALT: alanine aminotransferase, GGT: *γ*-glutamyl transpeptidase; tid: three times a day; qid: four times a day; UDCA: ursodeoxycholic acid.

Months of treatment and followup are absolute unless otherwise indicated.

*Includes one patient who also received prednisone but was not separable from the other 4 patients.

^†^Does not include two patients who received prednisone.

^††^Does not include a third patient who also received prednisolone and mizoribine.

^‡^Pediatric patients.

^‡‡^Original case report states there was dramatic improvement in patient's condition, including defervescence, return of appetite, and reduction of serum bilirubin, and after 2 weeks, becoming completely asymptomatic. Six months later, patient returned with clinical worsening, which again responded to metronidazole.
